# Improved model adaptation approach for recognition of reduced-frame-rate continuous speech

**DOI:** 10.1371/journal.pone.0206916

**Published:** 2018-11-07

**Authors:** Lee-Min Lee, Hoang-Hiep Le, Fu-Rong Jean

**Affiliations:** 1 Department of Electrical Engineering, Da-Yeh University, Dacun, Changhua, Taiwan; 2 Faculty of Electronics–Telecommunications, Saigon University, Ho Chi Minh City, Vietnam; 3 Department of Electrical Engineering, National Taipei University of Technology, Taipei, Taiwan; Northwestern University, UNITED STATES

## Abstract

In distributed speech recognition applications, the front-end device that stands for any handheld electronic device like smartphones and personal digital assistants (PDAs) captures the speech signal, extracts the speech features, and then sends the speech-feature vector sequence to the back-end server for decoding. Since the front-end mobile device has limited computation capacity, battery power and bandwidth, there exists a feasible strategy of reducing the frame rate of the speech-feature vector sequence to alleviate the drawback. Previously, we proposed a method for adjusting the transition probabilities of the hidden Markov model to enable it to address the degradation of recognition accuracy caused by the frame-rate mismatch between the input and the original model. The previous model adaptation method is referred to as the adapting-then-connecting approach that adapts each model individually and then connects the adapted models to form a word network for speech recognition. We have found that this model adaption approach introduces transitions that skip too many states and increase the number of insertion errors. In this study, we propose an improved model adaptation approach denoted as the connecting-then-adapting approach that first connects the individual models to form a word network and then adapts the connected network for speech recognition. This new approach calculates the transition matrix of a connected model, adapts the transition matrix of the connected model according to the frame rate, and then creates a transition arc for each transition probability. The new approach can better align the speech feature sequence with the states in the word network and therefore reduce the number of insertion errors. We conducted experiments to investigate the effectiveness of our new approach and analyzed the results with respect to insertion, deletion, and substitution errors. The experimental results indicate that the proposed new method obtains a better recognition rate than the old method.

## I. Introduction

In the era of instant wireless communication with intelligent client mobile devices, the human machine interface (HMI) based on automatic speech recognition (ASR) is becoming increasingly important. A client mobile device attributes the following properties: wireless access to the internet, a battery that provides limited power for a couple of days at most, small size that allows it to be carried in one hand, and a touch-screen interface for entering and displaying information. In speech recognition, a hidden Markov model (HMM) speech recognizer needs intense computation to fulfill the Viterbi decoding process. On the contrary, speech feature extraction consumes much less computation. Since the computation capacity of a mobile device is limited, low-complexity speech-feature extraction can be carried out by the client mobile device and high-complexity speech decoding can be performed by the backend powerful cloud server. This attractive client–server architecture is referred to as distributed speech recognition (DSR) [1–4]. Since under certain operating conditions the computational capacity, battery power, and transmission bandwidth resources of mobile devices may be very limited, there is a demand for the DSR system to work at a reduced frame rate. The apparent advantages of frame rate changes are not only minimizing computation in front-end devices but also reducing computation cost in the back-end server which allows it to serve more client users simultaneously under the same level of recognition accuracy when applying adaptation models [5].

The model parameters of a speech recognition server are typically trained from full frame rate (FFR) observation data. FFR observation data provide a sequence of speech feature vector that is carried out by a front-end algorithm. The front end algorithm consists of both framing and feature extraction processes. The former splits the speech samples into frames of constant length for simplifying block-wise processing of the speech signal, and the latter calculates a compact parametric spectrum representation of speech features that are intensely relevant for speech recognition. If the model parameters are directly applied to the recognition of reduced frame rate (RFR) speech, the performance will significantly degrade because the frame rates of the front-end feature sequence and back-end model are mismatched. In our previous study [5], the experimental result shows that using models trained from FFR clean data, the word accuracy for FFR and HFR clean data are 99.28% and 82.96%, respectively. There indeed exists a significantly performance degradation. In the past, several approaches have been proposed to compensate for the performance degradation caused by the frame-rate mismatch. Tan et al. [6] reconstructed the FFR data sequence by repeating each frame in the half frame rate (HFR) observation sequence, and then using the original FFR hidden Markov models (HMMs) to decode the reconstructed FFR feature sequence. The authors also suggested a multi-frame rate adaptation method, which allows the system to switch between the HFR and FFR. Linear and non-linear interpolation methods are also popular traditional approaches for reconstructing missing frames [7]. Instead of reconstructing an FFR feature sequence from the RFR one, we proposed a model adaptation method that adapts the state-transition probabilities of the FFR HMM to match the RFR of the input feature vector sequence [5, 8]. In that adaptation method, each model is individually adapted and then connected to form a word network for speech decoding (i.e., automatic speech recognition, ASR). However, this model adaptation approach creates transitions that skip too many states and increase the number of insertion errors. Subsequently, we proposed an improved model adaptation method that first connects the individual models to form a word network and then adapts the connected network for speech recognition, the concept of which we briefly outlined in a letter [9]. In this paper, we present our detailed formulation, implementation, experiments, and analysis of experimental results for this new model adaptation approach.

## II. Methods

### 2.1 Adaptation of hidden Markov models for isolated word speech at reduced frame rate

Most speech recognition systems are classified as either isolated or continuous. Isolated word recognition demands a short pause after each spoken word, whereas continuous speech recognition does not. Nowadays, the hidden Markov model [10] is one of the most popular and successful speech recognizers. A hidden Markov model consists of a finite set of states. Transitions among the states and observations generated in emitting states are governed by two sets of probabilities called state-transition probability distributions and observation symbol probability distributions, respectively. The state is not directly visible, but the observation dependent on the state is visible to an external observer. In our following explanation a particular matrix consists of elements of state-transition probability distribution that concerns the probability from a state to another state in a single step called the transition matrix.

Assume that **o**_1_,**o**_2_,⋯,**o**_*T*_ is the FFR observation sequence of an isolated word to be recognized and **o**_*D*_,**o**_2*D*_,⋯,**o**_*KD*_ is an RFR subsequence with a reduction factor of *D*. Typically, a speech recognizer calculates the likelihood score for each model to generate the word to be recognized. In an HMM, we let {0,1, 2, ⋯, *N+*1} denote the indices of the model states, in which states 0 and *N*+1 are the two special non-emitting starting and ending null states and states 1 through *N* are the emitting states. An emitting state produces a random output each time it is visited. The starting and ending non-emitting null states represent the end boundaries before the first and after the last observations, respectively. The set of parameters of an HMM includes the state-transition probabilities and state-output probability distributions. We use *Q*_*t*_ to denote the state of the system at time *t*, *a*_*ij*_ to denote the transition probability from state *i* to state *j*, and *b*_*j*_(**o**) to denote the probability density function for state *j* to produce the observation **o**. To calculate the probability that the FFR observation sequence is generated by a model λ, we can define a forward probability density function for the model to be in state *i* at time *t* and produce observations up to time *t*.

$$\alpha \left[i,t\right]=P\left(o_{1},o_{2},\cdots ,o_{t},Q_{t}=i|\lambda \right).$$ (1)

This forward function can be calculated recursively as follows:

$$\begin{array}{l} \alpha \left[j,t+1\right]=P\left(o_{1},o_{2},\cdots ,o_{t+1},Q_{t+1}=j|\lambda \right)\\ \;=\sum_{i=2}^{N+1}P\left(o_{1},o_{2},\cdots ,o_{t+1},Q_{t}=i,Q_{t+1}=j|\lambda \right)\\ \;=\sum_{i=2}^{N+1}P\left(o_{1},o_{2},\cdots ,o_{t},Q_{t}=i|\lambda \right)P\left(o_{t+1},Q_{t+1}=j|o_{1},o_{2},\cdots ,o_{t},Q_{t}=i,\lambda \right)\\ \;=\sum_{i=2}^{N+1}\alpha \left[i,t\right]P\left(Q_{t+1}=j|o_{1},o_{2},\cdots ,o_{t},Q_{t}=i,\lambda \right)P\left(o_{t+1}|o_{1},o_{2},\cdots ,o_{t},Q_{t}=i,Q_{t+1}=j,\lambda \right)\\ \;=\sum_{i=2}^{N+1}\alpha \left[i,t\right]a_{ij}b_{j}\left(o_{t+1}\right). \end{array}$$ (2)

We can calculate the probability of the FFR sequence as follows:

$$P\left(o_{1},o_{2},\cdots ,o_{T}|\lambda \right)=\sum_{i=1}^{N}P\left(o_{1},o_{2},\cdots ,o_{T},Q_{T}=i,Q_{T+1}=N+1|\lambda \right)=\sum_{i=1}^{N}\alpha \left[i,T\right]a_{i,N+1}.$$ (3)

To calculate the probability density function for the model to produce the RFR subsequence, we can define the following forward probability density function:

$$\alpha_{D}\left[i,k\right]=P\left(o_{D},o_{2D},\cdots ,o_{KD},Q_{kD}=i|\lambda \right).$$ (4)

**Fig 1:**
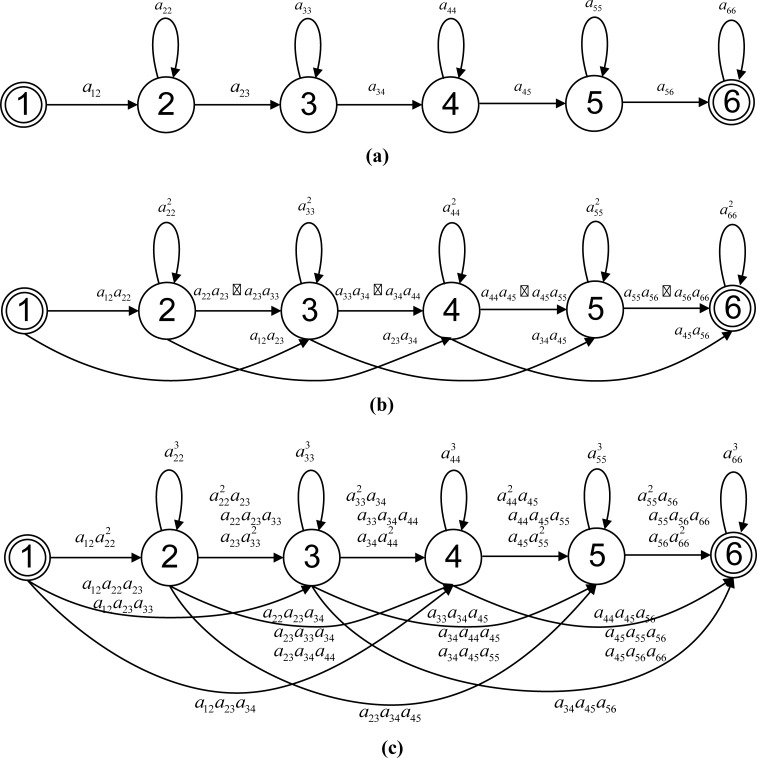
Topologies for original FFR HMM and corresponding adapted HMMs. (a) Original six-state HMM topology. (b) Adapted HMM topology for half frame rate (*D* = 2). (c) Adapted HMM topology for one-third frame rate (*D* = 3).

This forward function can be calculated as follows:

$$\begin{array}{l} \alpha_{D}\left[j,k+1\right]=P\left(o_{D},o_{2D},\cdots ,o_{(k+1)D},Q_{(k+1)D}=j|\lambda \right)\\ \;=\sum_{i=2}^{N+1}\sum_{i_{1}=2}^{N+1}\sum_{i_{2}=2}^{N+1}\cdots \sum_{i_{D-1}=2}^{N+1}P\left(\begin{array}{l} o_{D},o_{2D},\cdots ,o_{kD},o_{(k+1)D},\\ Q_{kD}=i,Q_{kD+1}=i_{1},\cdots ,Q_{kD+D-1}=i_{D-1},Q_{(k+1)D}=j \end{array}|\lambda \right)\\ \;=\sum_{i=2}^{N+1}\sum_{i_{1}=2}^{N+1}\sum_{i_{2}=2}^{N+1}\cdots \sum_{i_{D-1}=2}^{N+1}\left\{\begin{array}{l} P\left(o_{D},o_{2D},\cdots ,o_{kD},Q_{kD}=i|\lambda \right)\\ \times P\left(Q_{kD+1}=i_{1}|Q_{kD}=i,\lambda \right)\\ \times P\left(Q_{kD+2}=i_{2}|Q_{kD+1}=i_{1},\lambda \right)\\ \vdots \\ \times P\left(Q_{(k+1)D}=j|Q_{kD+D-1}=i_{D-1},\lambda \right)\\ \times P\left(o_{(k+1)D}|Q_{(k+1)D}=j,\lambda \right) \end{array}\right\}\\ \;=\sum_{i=2}^{N+1}\sum_{i_{1}=2}^{N+1}\sum_{i_{2}=2}^{N+1}\cdots \sum_{i_{D-1}=2}^{N+1}\left\{\alpha_{D}\left[i,k\right]a_{i,i_{1}}a_{i_{1},i_{2}}\cdots a_{i_{D-1},}{}_{j}b_{j}\left(o_{(k+1)D}\right)\right\}\\ \;=\sum_{i=2}^{N+1}\alpha_{D}\left[i,k\right]a_{i,j}^{\left(D\right)}b_{j}\left(o_{(k+1)D}\right), \end{array}$$ (5)

where

$$a_{i,j}^{\left(D\right)}=\sum_{i_{1}=2}^{N+1}\sum_{i_{2}=2}^{N+1}\cdots \sum_{i_{D-1}=2}^{N+1}a_{i,i_{1}}a_{i_{1},i_{2}}\cdots a_{i_{D-1},j}$$ (6)

is the transition probability from state *i* to state *j* for one step of the RFR sequence’s observation period (*D* times in one FFR observation period). Comparing Eqs (5) and (2), we can see that the forward function for the RFR sequence is computed using an equivalent HMM with the adapted transition probabilities given by Eq (6) and the unchanged state-output distributions. The RFR adaptation of the transition matrix of an HMM creates transitions that skip more states than those of the original model. For example, suppose that the original HMM topology is a six-state left-to-right HMM including the starting and ending null states without any skip transitions, as shown in **Fig 1A**. The corresponding adapted HMM for the HFR (*D* = 2) and one-third frame rate (*D* = 3) are quite different from the original HMM topology. A larger frame reduction factor will create more skip transitions over a greater length, as shown in **Fig 1B** and **1C**.

The adaptation of an HMM can also be illustrated using the power of its transition probability matrix. Let the following matrix:

$$A_{\mathrm{FFR}}=\left[a_{ij}\right]=\left[\begin{array}{cccccc} 0 & a_{12} & 0 & 0 & 0 & 0\\ 0 & a_{22} & a_{23} & 0 & 0 & 0\\ 0 & 0 & a_{33} & a_{34} & 0 & 0\\ 0 & 0 & 0 & a_{44} & a_{45} & 0\\ 0 & 0 & 0 & 0 & a_{55} & a_{56}\\ 0 & 0 & 0 & 0 & 0 & a_{66} \end{array}\right]$$ (7)

be the state-transition matrix of a six-state FFR HMM model, as shown in **Fig 1A**. Note that the sum of each row is equal to 1, and especially that *a*_12_ = *a*_66_ = 1. Since the observation period of the RFR subsequence is twice that of the original FFR sequence, the FFR HMM must go through two state-transition steps to get one RFR state-transition step. Therefore, the state-transition matrix for the RFR subsequence must be the square of the transition matrix for the FFR sequence, i.e.,:

$$A_{\mathrm{HFR}}=A_{\mathrm{FFR}}^{2}=\left[\begin{array}{cccccc} 0 & a_{12}a_{22} & a_{12}a_{23} & 0 & 0 & 0\\ 0 & a_{22}^{2} & a_{22}a_{23}+a_{23}a_{33} & 0 & 0 & 0\\ 0 & 0 & a_{33}^{2} & a_{33}a_{34}+a_{34}a_{44} & 0 & 0\\ 0 & 0 & 0 & a_{44}^{2} & a_{44}a_{45}+a_{45}a_{55} & 0\\ 0 & 0 & 0 & 0 & a_{55}^{2} & a_{55}a_{56}+a_{56}a_{66}\\ 0 & 0 & 0 & 0 & 0 & a_{66}^{2} \end{array}\right].$$ (8)

The non-zero entries of the above matrix are simply the links in **Fig 1B**. We can easily generalize the state-transition matrix for the one-third frame rate observation sequence and obtain $A_{(1/3)\mathrm{FR}}=\left[a_{ij}^{\left(3\right)}\right]=A_{\mathrm{FFR}}^{3}$, which leads to the adapted HMM topology for the one-third frame rate data shown in **Fig 1C**. For a subsequence with a frame reduction factor of *D*, one state-transition step between two consecutive observations is equivalent to *D* state-transition steps in the original FFR model. Therefore, the transition matrix for the RFR subsequence should be the *D*^*th*^ power of the FFR transition matrix.

### 2.2 Adaptation of hidden Markov models for recognition of continuous speech with reduced frame rate

**Fig 2:**
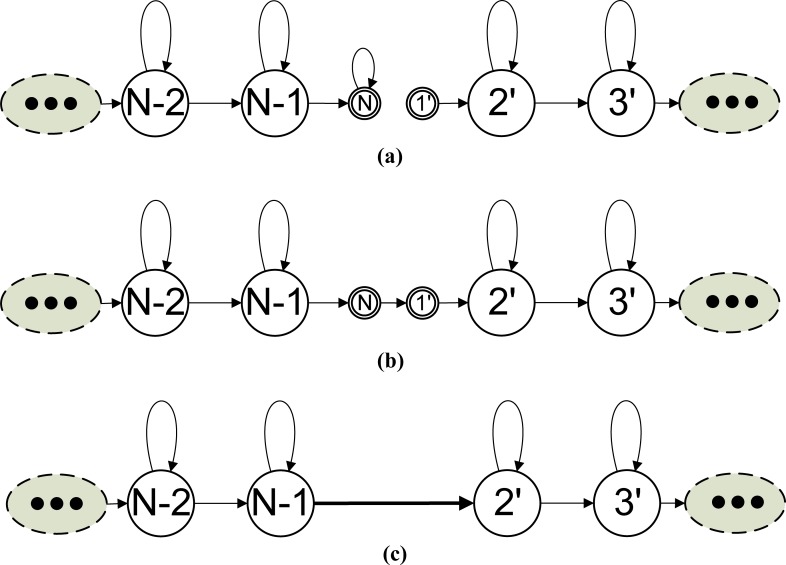
Procedure for connecting two HMMs. (a) Two individual HMMs. (b) The two individual HMMs are concatenated by diverting the target of the self-transition from the ending null state of the first model to the starting null state of the following model. (c) The equivalent HMM of (b) as the two passing-through null states are removed.

In continuous speech recognition, HMMs must be connected to form a word network for decoding. A word network defines the sequence of words that can be recognized. When an HMM is connected to the following HMM, the self-transition link of the null ending state of the first HMM must be diverted to the starting null state of the next model, since the ending null state of an HMM represents the time after the last observation of that model. After the self-transition link of the ending null state of the previous HMM is diverted to connect to the starting null state of the next HMM, we can remove these two passing-through null states. **Fig 2** illustrates this connection procedure.

**Fig 3:**
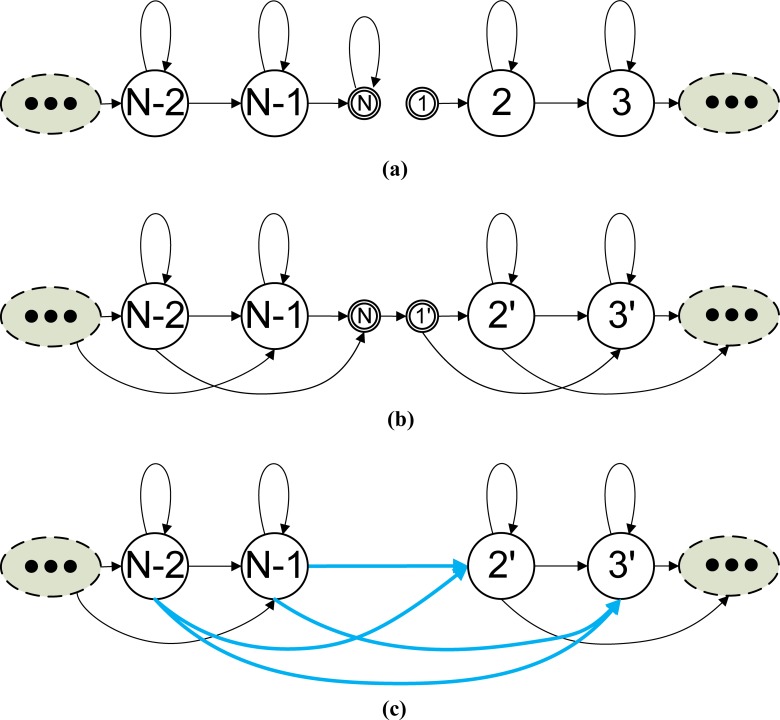
Illustration of the adapting-then-connecting approach for HFR adaptation of HMMs. (a) Two individual original FFR HMMs. (b) The two individually adapted HMMs are concatenated. (c) The equivalent HMM of (b) when the passing-through null states are removed.

There are two possible approaches for adjusting the transition probabilities of a connected word network for recognizing the RFR observation sequence. The first approach is an adapting-then-connecting approach, as shown in **Fig 3**, in which individual word models are adapted first, according to the RFR factor *D*, and are then connected to form the word network for RFR speech decoding. The elements of the transition matrix of the adapted combined model include the word-internal state transitions and word-outgoing state transitions. The latter are transitions from the states of the first model to those of the second model. In **Fig 3A**, we have two FFR HMMs with left-to-right topology and without any state-skipping transitions. In **Fig 3B**, the two HMMs are first individually adapted and are then connected by diverting the self-loop transition of the ending null state of the first HMM to the starting null state of the second HMM. **Fig 3C** shows the equivalent HMM of **Fig 3B** as the two passing-through null states are removed. If a state can directly jump to the ending null state of a model, it can also jump to the states to which the following HMM’s starting null state can jump. From **Fig 3B** and **3C**, we can see that the transition from state (*N*-2) to state 3' skips two states, which is unreasonable for HFR speech. Therefore, the RFR HMM adaptation approach creates transitions that skip more states than an actual RFR model can jump over. In speech recognition, an insertion error is when a word is recognized but in fact none was spoken. These excessive-jump state-transition links hinder the alignment of the RFR speech sequence with the connected adapted HMMs and consequently increase the number of insertion errors.

**Fig 4:**
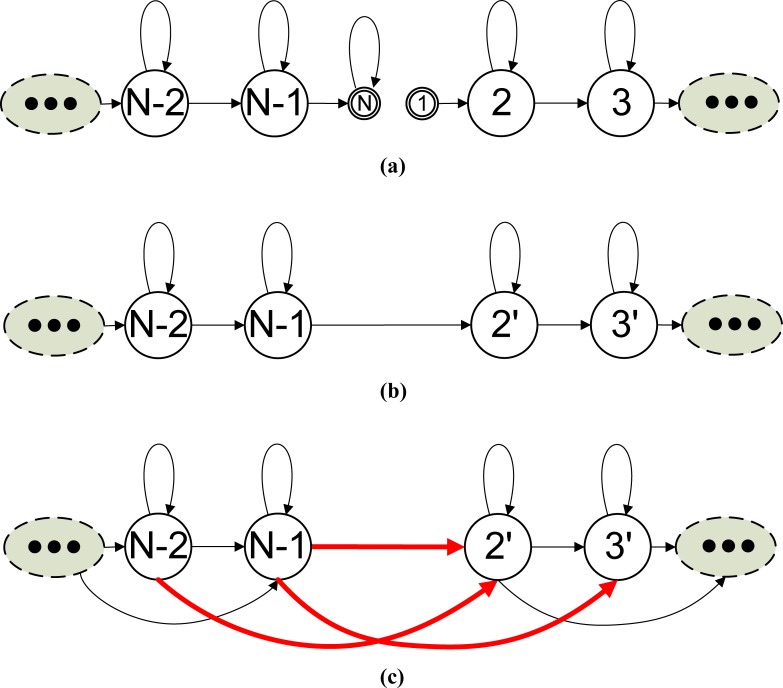
**Illustration of the connecting-then-adapting approach for HFR adaptation of HMMs.** (a) Two individual original FFR HMMs. (b) The two individual HMMs are concatenated and then the passing-through null states are removed. (c) The adapted combined HMM.

In contrast to the adapting-then-connecting order in the first approach, the second approach uses a connecting-then-adapting strategy to avoid creating links that skip too many states. In this approach, the transition probabilities from the states of an HMM to the states of a directly following HMM are determined first by connecting the two models to form a combined model, as shown in **Fig 4A and 4B**, and then the combined HMM is adapted according to the frame rate reduction factor *D* to fit the RFR speech, as shown in **Fig 4C**. We can see that there are no excessive-jump transitions in **Fig 3C** and all the transitions can skip at most one state in the HFR adaptation case. This connecting-then-adapting approach is more accurate since it avoids the problem of skipping too many states and alleviates the insertion-prone problem. In this adaptation approach, the destinations and associated probabilities that an emitting state may reach at the next RFR observation time are exactly the same as that it may reach at the next *D* FFR observation time in the FFR network. That is, at the same time, the (prior) probability of the emitting states in the RFR network is the same as that in the FFR network.

### 2.3 Design and implementation of a decoder for adapted hidden Markov model network

In a connected-digit recognition system, digital, silence, and short-pause models are connected to form a word loop network and the Viterbi algorithm is used to find the best path for the feature vector sequence of a test utterance to move through the network. A traditional network of HMMs is characterized by each model in the connected network having only a single entrance and a single exit. This forces the emitting state of an HMM to make transitions only to a following HMM’s emitting states by going through its exit null state and the entrance null state of the following HMM. This characteristic lessens the burden in designing the decoder program and almost all publicly available HMM toolkits have this feature. After applying our proposed connecting-then-adapting method to the original FFR word network, the adapted RFR network no longer has the single entrance/exit characteristic and an emitting state can jump directly to an emitting state of a following HMM. Moreover, there are transitions that can jump over a series of HMMs with tee-transition, which is a transition link from the entrance null state to the exit null state of an HMM, to an emitting state of an HMM following this series. Since almost all publicly available HMM toolkits rely on the single entrance/exit characteristic, they cannot be directly applied to our new adapted network and we had to design and implement a new connected-digit decoder from scratch.

**Fig 5:**
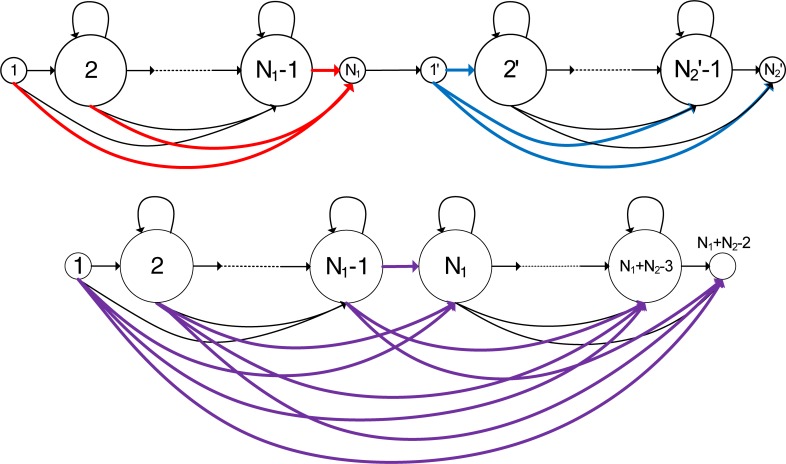
Two HMMs are concatenated to form a combined HMM.

In our design, we represent an HMM by a data structure that contains the FFR transition matrix and sub-data structures for each of its emitting states. The data structure for an emitting state contains both the parameters for its output probability distribution and transition links to each target emitting state that it can reach at the next observation time, including word-internal links and word-outgoing links that point to the data structure of an emitting state in another HMM. The data structure for a transition link includes a pointer to the target emitting state and the associated transition probability to that state. When the HMMs are connected to form a network, we must first create the transition links for each emitting state. To do so, we first create transition links to the emitting states of the same HMM based on the transition matrix of that HMM. Next, we create transition links to the emitting states of other HMMs. If we suppose two HMMs with *N*_1_ and *N*_2_ states (including both starting and ending null states) are concatenated in a network, we can compose a combined HMM with (*N*_1_ + *N*_2_−2) states to create links from the first HMM to the second HMM. **Fig 5** shows how two HMMs can be concatenated to form a combined HMM. The links to the ending null state of the first HMM are highlighted by the thick red lines. These links can reach states in the second HMM via the links beginning with the starting null state of the second HMM, which are highlighted by the thick blue lines. When the two models are combined, the two passing-through null states in between can be removed, and the total number of states in the combined model becomes (*N*_1_ + *N*_2_-2). In the figure, the concatenation of each pair of red and blue links is indicated by the purple links, which represent transitions from the first HMM to the second HMM.

We can compute the transition probability for the links from the first HMM to the second HMM as follows. Let the transition probability matrix of the two HMMs be

$$A_{\mathrm{HMM}1}={\left[\alpha_{ij}\right]}_{N_{1}\times N_{1}}={\left[\begin{array}{ccc} \alpha_{11}^{} & \cdots & \alpha_{1N_{1}}^{}\\ \vdots & \ddots & \vdots \\ \alpha_{N_{1}1}^{} & \cdots & \alpha_{N_{1}N_{1}}^{} \end{array}\right]}_{N_{1}\times N_{1}}$$ (9)

and

$$A_{\mathrm{HMM}2}={\left[\beta_{ij}\right]}_{N_{2}\times N_{2}}={\left[\begin{array}{ccc} \beta_{11}^{} & \cdots & \beta_{1N_{2}}^{}\\ \vdots & \ddots & \vdots \\ \beta_{N_{2}1}^{} & \cdots & \beta_{N_{2}N_{2}}^{} \end{array}\right]}_{N_{2}\times N_{2}},$$ (10)

respectively. Here, the last column elements of **A**_HMM1_ and the first row elements of **A**_HMM2_ correspond to the red and blue arcs in **Fig 5**, respectively. The transition probability matrix of the combined model becomes the following:

$$\begin{array}{l} A_{\mathrm{HMM}1+\mathrm{HMM}2}={\left[\gamma_{ij}\right]}_{\left(N_{1}+N_{2}-2\right)\times \left(N_{1}+N_{2}-2\right)}\\ =\left[\begin{array}{ccccccc} \alpha_{11}^{} & \cdots & \alpha_{1\left(N_{1}-1\right)}^{} & \underset{\gamma_{1N_{1}}^{}}{\underset{︸}{\alpha_{1N_{1}}^{}\times \beta_{12}^{}}} & \cdots & \underset{\gamma_{1\left(N_{1}+N_{2}-3\right)}^{}}{\underset{︸}{\alpha_{1N_{1}}^{}\times \beta_{1\left(N_{2}-1\right)}^{}}} & \underset{\gamma_{1\left(N_{1}+N_{2}-2\right)}^{}}{\underset{︸}{\alpha_{1N_{1}}^{}\times \beta_{1N_{2}}^{}}}\\ \alpha_{21} & \cdots & \alpha_{2\left(N_{1}-1\right)}^{} & \underset{\gamma_{2N_{1}}^{}}{\underset{︸}{\alpha_{2N_{1}}\times \beta_{12}^{}}} & \cdots & \underset{\gamma_{2\left(N_{1}+N_{2}-3\right)}^{}}{\underset{︸}{\alpha_{2N_{1}}^{}\times \beta_{1\left(N_{2}-1\right)}^{}}} & \underset{\gamma_{2\left(N_{1}+N_{2}-2\right)}^{}}{\underset{︸}{\alpha_{2N_{1}}^{}\times \beta_{1N_{2}}^{}}}\\ \vdots & \ddots & \vdots & \vdots & \ddots & \vdots & \vdots \\ \alpha_{\left(N_{1}-1\right)1}^{} & \cdots & \alpha_{\left(N_{1}-1\right)\left(N_{1}-1\right)}^{} & \underset{\gamma_{\left(N_{1}-1\right)N1}^{}}{\underset{︸}{\alpha_{\left(N_{1}-1\right)N_{1}}^{}\times \beta_{12}^{}}} & \cdots & \underset{\gamma_{\left(N_{1}-1\right)\left(N_{1}+N_{2}-3\right)}^{}}{\underset{︸}{\alpha_{\left(N_{1}-1\right)N_{1}}^{}\times \beta_{1\left(N_{2}-1\right)}^{}}} & \underset{\gamma_{\left(N_{1}-1\right)\left(N_{1}+N_{2}-2\right)}^{}}{\underset{︸}{\alpha_{\left(N_{1}-1\right)N_{1}}^{}\times \beta_{1N_{2}}^{}}}\\ 0 & \cdots & 0 & \beta_{22}^{} & \beta_{23}^{} & \cdots & \beta_{2N_{2}}^{}\\ \vdots & \ddots & \vdots & \vdots & \vdots & \ddots & \vdots \\ 0 & \cdots & 0 & \beta_{N_{2}2}^{} & \beta_{N_{2}3}^{} & \cdots & \beta_{N_{2}N_{2}}^{} \end{array}\right] \end{array}.$$ (11)

The upper left and lower right submatrixes of the transition probability matrix for the combined model come from the upper left (*N*_1_−1)×(*N*_1_−1) submatrix of the first matrix, and the lower right (*N*_2_−1)×(*N*_2_−1) submatrix of the second matrix, respectively. The elements in the upper right (*N*_1_−1)×(*N*_2_−1) submatrix represent the transitions from the first HMM to the second HMM and the transition probability from state *i* of the first HMM to state *j* of the second HMM is given by $\alpha_{iN_{1}}\times \beta_{1j}$. We can then create transition links from the emitting states of the first HMM to the emitting states of the second HMM using the non-zero elements in this upper right submatrix of (11), except the first row and the last column of that submatrix (because they represent transitions either from or to a null state). Note that the non-zero element in the last column of the upper right submatrix of (11) represents a transition from the first HMM to the ending null state of the second HMM and therefore it can also reach the emitting states of a third HMM if the third HMM is connected to the end of the second HMM. In that case, we must create links from the first HMM to the third HMM by combining the three HMMs, computing the transition matrix of the combined HMM, and then creating transition links from each non-zero element in the combined transition matrix that is associated with a transition from the first HMM to the third HMM. This process continues until we create all the transition links that correspond to all the targets that the emitting states in the first HMM can reach in one observation time step.

**Fig 6:**
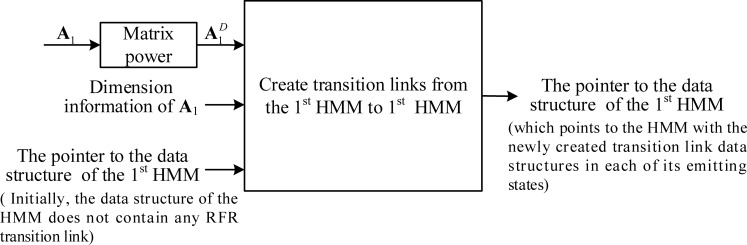
The creation of model internal transition links for frame rate reduction factor *D*.

In the adapting-then-connecting approach, we first adapt the transition matrix of each HMM to the matrix power of the frame-rate reducing factor *D*, and then create transition-link data structures for all possible destinations that an emitting state can reach in the next observation time (of the RFR). **Fig 6** illustrates the process for creating the model’s internal transition links. For convenience, let the HMM in **Fig 6** be denoted as the first HMM and its transition matrix be denoted by ***A***_1_. Initially, the data structure for the HMM contains its FFR transition matrix but none of its emitting states contain a transition-link data structure for the frame-rate reduction factor *D*. In each of the HMM’s emitting states, we then create a transition-link data structure from each of the non-zero elements in the corresponding row $A_{1}^{D}$, excepting the elements in the last column.

**Fig 7:**
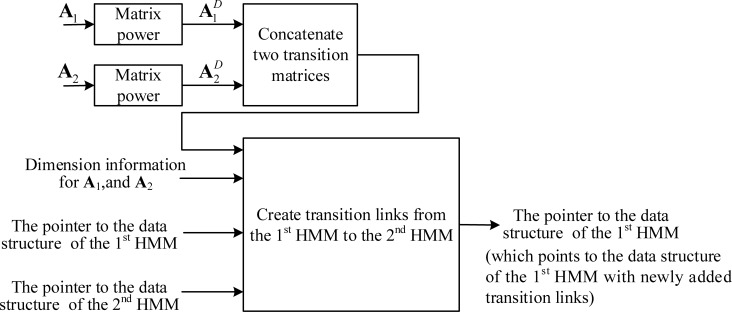
Creation of transition links from the 1^st^ HMM to the 2^nd^ HMM for frame-rate reduction factor *D* using the adapting-then-connecting approach.

After the model internal transition links are created, we must create transition links to all the HMMs that immediately follow the first HMM. Let an HMM immediately following the first HMM be denoted as the second HMM. **Fig 7** shows the process of creating transition-link data structures from the first HMM to the second HMM. As shown in the figure, we use the dimension information of the two matrixes to find the elements in the concatenated matrix that represent the transition probability from the emitting states of the first HMM to the second HMM and create the corresponding transition-link data structures in the emitting states of the first HMM. Let *N*_1_, *N*_2_ be the dimension for the transition matrix of the 1^st^ and 2^nd^ HMMs, respectively. The non-zero elements in the last *N*_2_ columns of the 2^nd^ to the *N*_1_-th rows of the concatenated matrix are the transition probabilities from the emitting state of the 1^st^ HMM to the 2^nd^ HMM. Using these probabilities and the data structure pointers of the two HMMs, we can create transition-link data structures in the emitting states of the 1^st^ HMM to point to the emitting states of the 2^nd^ HMM. Note that there may be several HMMs directly connected to the end of the first HMM, and we must create transition links in the first HMM to point to all the directly following HMMs.

**Fig 8:**
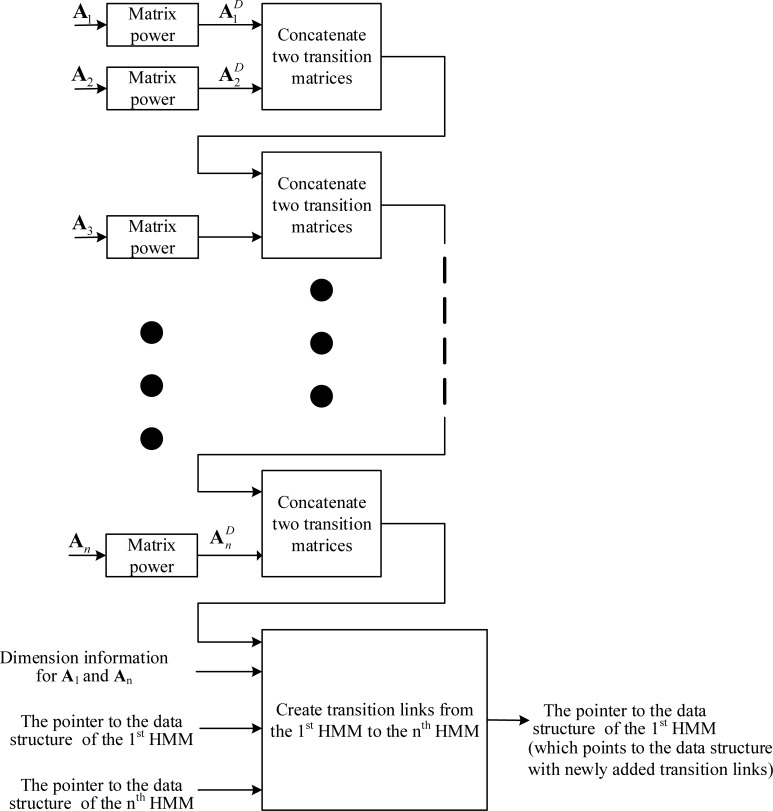
The creation of transition links from the 1^st^ HMM to the *n*^th^ HMM for frame rate reduction factor *D* using the adapting-then-connecting approach.

If the adapted transition matrix of the second model includes a tee transition, we must create RFR transition links from the first model to the models that directly follow the second model. This process of creating transition links continues until links are created to all possible destinations that an emitting state can reach at the next observation time (*D* times of the FFR observation period). **Fig 8** illustrates the process of creating transition-link data structures from the 1^st^ HMM to the *n*^th^ HMM.

**Fig 9:**
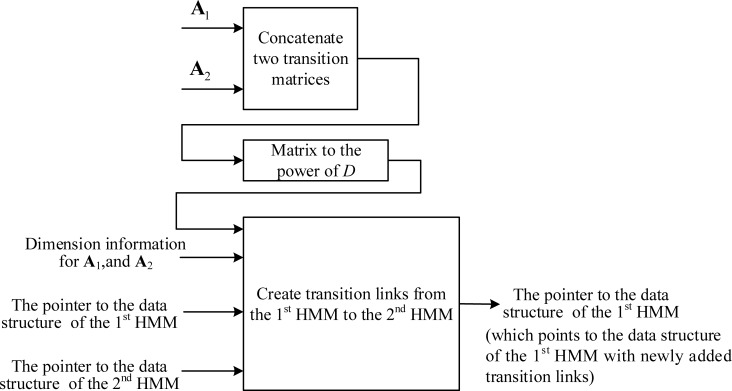
Creation of transition links from the 1^st^ HMM to the 2^nd^ HMM for frame-rate reduction factor *D* using the connecting-then-adapting approach.

**Fig 10:**
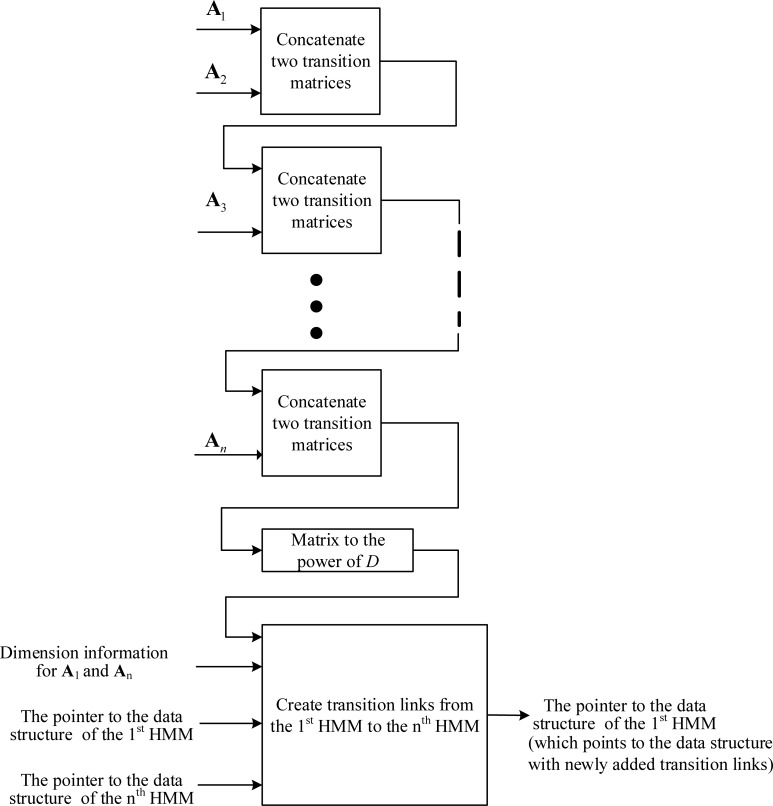
Creation of transition links from the 1^st^ HMM to the n^th^ HMM for frame-rate reduction factor *D* using the connecting-then-adapting approach.

In the connecting-then-adapting approach, we created transition links for RFR speech by first computing the transition probability matrix of the connected model, raising it to the power of the frame-rate reduction factor *D*, and then creating links using the adapted matrix. The process of creating model internal transition links was the same as that shown in **Fig 6**. **Fig 9** illustrates the process for creating transition-link data structures from an HMM (refered to as the 1^st^ HMM) to a directly following HMM (refered to as the 2^nd^ HMM). We must create transition-link data structures for all the destinations that an emitting state can reach at the next RFR observation time. **Fig 10** illustrates the process for creating transition-link data structures from the 1^st^ HMM to the *n*^th^ HMM.

**Fig 11:**
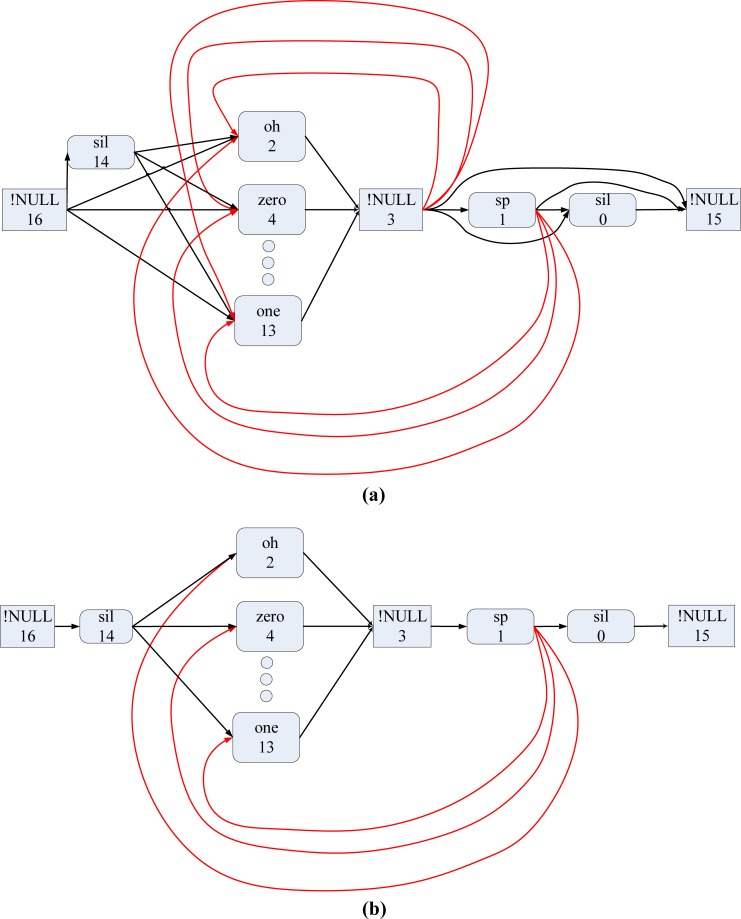
Original and simplified word loop networks. (a) Original Aurora2 word loop network. (b) Our simplified word loop network.

**Fig 12:**
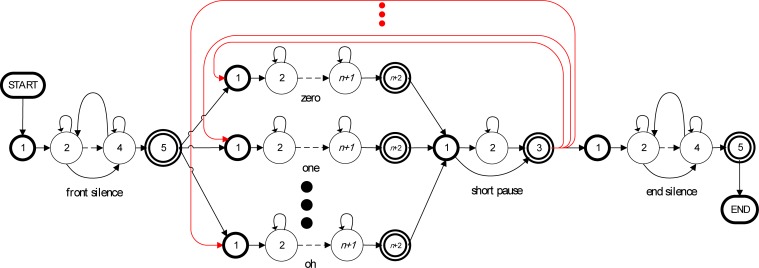
Detailed network of our connected-digit recognition system.

In this study, we used the Aurora2 [11] database in our experiments to evaluate the performance of the adaptation methods. We simplified the word loop network provided by the Aurora2 database to reduce the programming complexity without sacrificing recognition accuracy. **Fig 11A** and **11B** show the original Aurora2 word loop network and our simplified version, respectively. In **Fig 12**, we have expanded each word-level model to show the details of its HMM structure. The whole network contains one system start node, one system end node, and fourteen HMMs comprising a front silence, an end silence, a short pause (SP) and 11 English digits (zero, ‘oh’, one, two, …, and nine). The front and end silence models share the same model parameter set.

We implemented a modified token-passing algorithm [12] to decode FFR and RFR speech. In our design, a token represents a candidate partial path and its associated likelihood score. The path information of a token includes state-level and word-level paths. A path is represented and implemented as a string of states or digits depending on the path level. At every observation time *t*, each emitting state holds a token that represents the best subpath that reaches that state at that time. A null state holds no token and represents a place where a token can pass through instantly. Initially, each emitting state holds a token with a negative infinite score and empty paths, and the system-start node holds a token with score equal to 0 and empty paths. For each new observation time, in each emitting state, the stored token is propagated along the state’s transition links to its destination. The system-start state propagates its token only at the first observation time. When a token is propagated along a link, we add the token’s score by the amount of the link’s log probability and append the new node to the token’s path. When the propagation is through a word-outgoing link, we also update the word-level path. In each destination state, we collect incoming tokens and select the one with the maximum score, add its score to the log probability that the observation was generated by the destination state, and then update the stored token with this maximum score token. We designate a state as the system-end node of the whole network for the purpose of collecting tokens after the last observation is processed. Finally, we select the token with the highest score in the system-end node and retrieve its word-level path as the recognition result.

## III. Experiments and results

In the experiments, we used the Aurora2 database to investigate the effectiveness of the HMM adaptation methods for the task of speaker-independent connected-digit recognition in clean and noisy environments.

### 3.1 Speech feature extraction, model structure and training methods

We used 12 mel-frequency cepstral coefficients (MFCCs) and one log energy as the static feature vector. We set the frame length and frame shift times for the FFR observation sequence to 25 ms and 10 ms, respectively. The dynamic feature vector was composed of delta and acceleration coefficients of the static feature sequence and the feature vector for each frame of speech consisted of a total of 39-dimension speech features. The processing details with respect to feature extraction and expansion were exactly the same as those provided by the Aurora2 database. In accordance with the recommendations of the European Telecommunication Standards Institute (ETSI), we transmitted only the static feature to the client and then we appended the dynamic feature after the static feature was received at the recognition server. We modeled each digit using an HMM with 16 emitting states, modeled silence using an HMM with three emitting states, and modeled SPs using an HMM with a single emitting state. The emitting state of the short-pause model and the middle state of the silence model shared the same state-output probability distribution. The SP had a tee transition from the null start state to the null end state so that it could be skipped when there is no pause between two digits. We used the Gaussian mixture distribution for the output of the emitting states. The number of mixture components for states in the silence model (and hence the short-pause model) and digital models were eight and four, respectively. We used the HTK Toolkit [10] to train the FFR speech model. We prepared two sets of FFR models, one of which was trained using the clean training condition and the other using the multi-training condition.

### 3.2 Recognition for RFR connected word speech

We investigated and compared the performances of the adapting-then-connecting and connecting-then-adapting approaches with respect to speech recognition of RFR speech. We tested the two model adaptation methods for their recognition of clean and noisy test data at several SNR levels from 0 dB to 20 dB in 5-dB steps. An ETSI repetition concealment method for recognition of RFR speech is also included for comparison [13]. **Table 1** shows the word accuracies of the ETSI repetition concealment method and the two adaptation models in various conditions, in which we can see that the connecting-then-adapting approach obtains slightly better accuracy than the adapting-then-connecting approach for *D* = 2, 3, and 4. **Table 1** also includes the word accuracy at the original frame rate (for *D* = 1) for allowing an assessment about the word accuracy degradation in terms of frame rate reduction. We can see that for multi-condition training the word accuracies for the ETSI repetition concealment method and the two adaptation models all are slightly worse than that of FFR data. Though the adapting-then-connecting approach performs the worst, the word accuracy degradation is limited within 0.97%, 2.52% and 4.58% (in average) for *D* = 2, 3 and 4, respectively.

**Table 1:** Word accuracy for FFR data and for the ETSI repetition and the two adaptation approaches on RFR data at various SNR levels in models based on clean and multi-condition training data.

Frame rate reduction factor *D*	SNR	clean condition training			multi-condition training		
		ETSI repetition	adapting-then-connecting	connecting-then-adapting	ETSI repetition	adapting-then-connecting	connecting-then-adapting
**1** **(FFR)**	**Clean**	99.42			99.05		
	**20 dB**	95.40			98.45		
	**15 dB**	85.21			97.69		
	**10 dB**	64.59			95.34		
	**5 dB**	37.71			89.53		
	**0 dB**	17.21			65.53		
	**Average**	**66.59**			**90.93**		
**2**	**Clean**	99.37	99.41	99.42	98.99	98.98	98.98
	**20 dB**	94.56	94.69	94.97	98.29	98.16	98.27
	**15 dB**	83.18	82.51	83.34	97.42	97.44	97.41
	**10 dB**	61.78	59.54	60.95	95.2	95.25	95.20
	**5 dB**	35.42	31.29	33.09	88.33	87.96	88.08
	**0 dB**	15.45	12.43	13.70	63.52	62.00	61.97
	**Average**	**64.96**	**63.31**	**64.25**	**90.29**	**89.97**	**89.99**
**3**	**Clean**	99.26	99.19	99.26	98.87	98.83	98.91
	**20 dB**	93.48	93.36	93.83	98.12	98.03	98.16
	**15 dB**	81.64	79.86	81.00	97.25	96.95	97.08
	**10 dB**	59.31	54.46	56.60	94.5	94.30	94.29
	**5 dB**	32.67	24.19	26.89	86.53	85.42	85.55
	**0 dB**	12.93	6.87	8.59	59.46	56.94	57.25
	**Average**	**63.22**	**59.66**	**61.03**	**89.12**	**88.41**	**88.54**
**4**	**Clean**	99.12	98.93	98.89	98.77	98.62	98.70
	**20 dB**	92.57	92.95	93.42	97.85	97.51	97.61
	**15 dB**	79.43	78.46	80.23	96.56	96.26	96.29
	**10 dB**	56.09	51.19	54.92	93.07	92.73	92.74
	**5 dB**	29.43	21.73	27.18	83.61	82.23	82.07
	**0 dB**	10.97	5.81	10.21	55.53	50.73	50.75
	**Average**	**61.27**	**58.18**	**60.81**	**87.57**	**86.35**	**86.36**

**Table 2** lists the insertion error rate for various conditions, in which we can see that the connecting-then-adapting approach had a lower insertion error rate than the adapting-then-adapting approach, as expected.

**Table 2:** Insertion error rate for the ETSI repetition and the two adaptation approaches.

Frame rate reduction factor *D*	SNR	clean condition training			multi-condition training		
		ETSI repetition	adapting-then-connecting	connecting-then-adapting	ETSI repetition	adapting-then-connecting	connecting-then-adapting
**2**	**Clean**	0.07	0.12	0.05	0.10	0.13	0.11
	**20 dB**	3.48	3.41	3.04	0.24	0.33	0.02
	**15 dB**	11.20	12.24	11.38	0.49	0.52	0.50
	**10 dB**	21.47	24.48	22.94	1.01	0.94	0.84
	**5 dB**	25.91	30.45	28.47	1.75	1.58	1.41
	**0 dB**	18.34	21.30	19.71	3.02	2.67	2.43
	**Average**	**13.41**	**15.33**	**14.27**	**1.10**	**1.03**	**0.88**
**3**	**Clean**	0.08	0.18	0.10	0.11	0.20	0.14
	**20 dB**	4.07	4.37	3.81	0.20	0.40	0.27
	**15 dB**	11.60	13.94	12.69	0.53	0.61	0.50
	**10 dB**	21.69	27.68	25.48	1.10	0.99	0.82
	**5 dB**	25.69	35.53	32.19	1.99	1.93	1.64
	**0 dB**	19.23	27.01	24.41	3.48	3.41	2.70
	**Average**	**13.72**	**18.12**	**16.45**	**1.24**	**1.26**	**1.01**
**4**	**Clean**	0.09	0.24	0.11	0.09	0.23	0.12
	**20 dB**	4.08	3.66	3.20	0.18	0.40	0.26
	**15 dB**	11.99	13.56	11.90	0.66	0.61	0.45
	**10 dB**	21.84	28.09	24.09	1.27	0.92	0.71
	**5 dB**	25.30	34.63	28.37	2.37	1.78	1.35
	**0 dB**	19.31	24.85	19.53	4.25	2.63	2.30
	**Average**	**13.77**	**17.50**	**14.53**	**1.47**	**1.09**	**0.86**

**Table 3** lists the deletion error rate for various conditions, in which we can see that although the connecting-then-adapting approach can reduce the insertion error rate, it can also increase the number of deletion errors. Some trade-off between the resulting insertion and deletion errors is inevitable, since the new approach puts a stricter constraint on the minimum length of a digit and can force a very rapid utterance to be aligned with fewer digits than it should.

**Table 3:** Deletion error rate for the ETSI repetition and the two adaptation approaches.

Frame rate reduction factor *D*	SNR	clean condition training			multi-condition training		
		ETSI repetition	adapting-then-connecting	connecting-then-adapting	ETSI repetition	adapting-then-connecting	connecting-then-adapting
**2**	**Clean**	0.23	0.19	0.22	0.34	0.27	0.33
	**20 dB**	0.35	0.32	0.41	0.31	0.26	0.31
	**15 dB**	0.78	0.63	0.67	0.37	0.33	0.37
	**10 dB**	2.74	1.94	2.11	0.86	0.78	0.91
	**5 dB**	8.25	5.77	6.27	3.43	3.89	3.99
	**0 dB**	20.79	19.70	20.76	19.75	22.49	22.76
	**Average**	**5.52**	**4.76**	**5.07**	**4.18**	**4.67**	**4.78**
**3**	**Clean**	0.26	0.20	0.23	0.36	0.24	0.30
	**20 dB**	0.43	0.34	0.43	0.34	0.19	0.26
	**15 dB**	1.05	0.54	0.74	0.43	0.36	0.42
	**10 dB**	3.44	1.67	1.89	0.90	0.83	0.97
	**5 dB**	9.18	4.97	5.41	3.88	4.58	4.83
	**0 dB**	19.34	15.96	16.84	20.65	24.85	25.20
	**Average**	**5.62**	**3.94**	**4.26**	**4.43**	**5.18**	**5.33**
**4**	**Clean**	0.28	0.23	0.37	0.42	0.25	0.40
	**20 dB**	0.67	0.47	0.63	0.43	0.27	0.41
	**15 dB**	1.46	0.82	1.01	0.55	0.44	0.63
	**10 dB**	3.96	2.12	2.47	1.12	1.16	1.49
	**5 dB**	9.85	5.55	6.78	4.53	6.31	6.73
	**0 dB**	18.52	17.81	20.76	21.52	31.54	31.99
	**Average**	**5.79**	**4.50**	**5.34**	**4.76**	**6.66**	**6.94**

**Table 4** lists the substitution error rate for various conditions, from which we can see that the substitution error rates of these two adaptation approaches are very similar.

**Table 4:** Substitution error rate for the ETSI repetition and the two adaptation approaches.

Frame rate reduction factor *D*	SNR	clean condition training			multi-condition training		
		ETSI repetition	adapting-then-connecting	connecting-then-adapting	ETSI repetition	adapting-then-connecting	connecting-then-adapting
**2**	**Clean**	0.33	0.28	0.31	0.57	0.62	0.57
	**20 dB**	1.61	1.58	1.57	1.16	1.26	1.18
	**15 dB**	4.84	4.62	4.61	1.72	1.71	1.73
	**10 dB**	14.01	14.05	14.00	2.94	3.03	3.05
	**5 dB**	30.42	32.49	32.17	6.50	6.58	6.53
	**0 dB**	45.41	46.57	45.84	13.71	12.84	12.85
	**Average**	**16.10**	**16.60**	**16.42**	**4.43**	**4.34**	**4.32**
**3**	**Clean**	0.40	0.43	0.41	0.66	0.73	0.65
	**20 dB**	2.02	1.94	1.93	1.34	1.37	1.32
	**15 dB**	5.72	5.66	5.57	1.79	2.07	1.99
	**10 dB**	15.56	16.20	16.03	3.50	3.88	3.92
	**5 dB**	32.46	35.31	35.51	7.60	8.07	7.98
	**0 dB**	48.51	50.16	50.16	16.41	14.80	14.85
	**Average**	**17.44**	**18.28**	**18.27**	**5.21**	**5.16**	**5.12**
**4**	**Clean**	0.50	0.61	0.62	0.73	0.90	0.79
	**20 dB**	2.69	2.92	2.75	1.54	1.82	1.72
	**15 dB**	7.13	7.16	6.86	2.23	2.69	2.62
	**10 dB**	18.12	18.60	18.53	4.55	5.19	5.06
	**5 dB**	35.43	38.09	37.68	9.49	9.68	9.84
	**0 dB**	51.20	51.53	49.50	18.71	15.10	14.96
	**Average**	**19.18**	**19.82**	**19.32**	**6.21**	**5.90**	**5.83**

**Fig 13:**
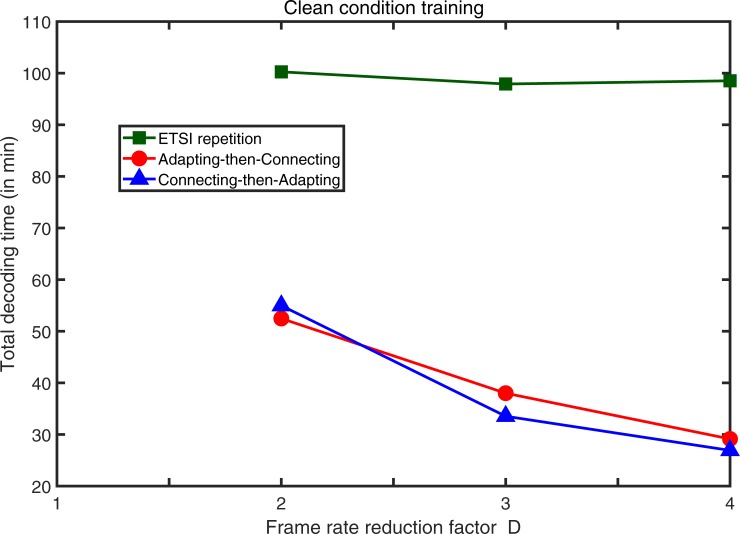
The total decoding time of all three test data sets of AURORA 2 with clean condition training for the ETSI repetition and the two adaptation approaches.

**Fig 14:**
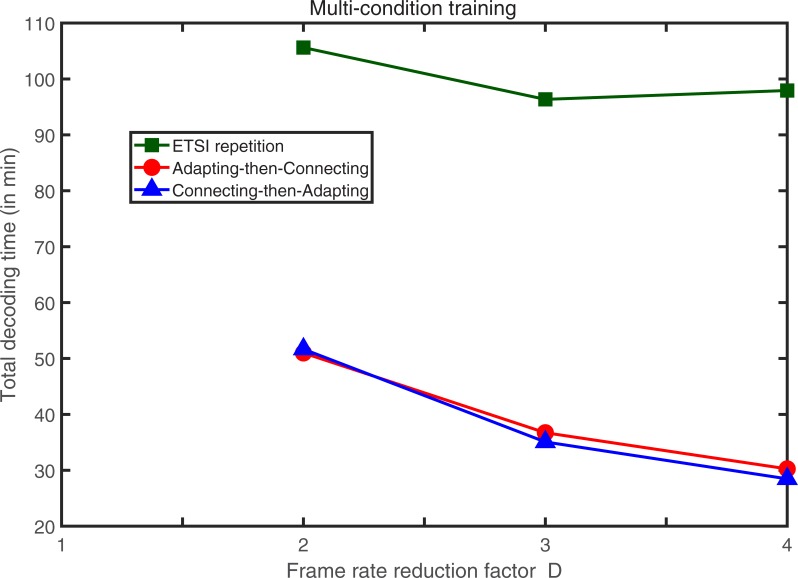
The total decoding time of all three test data sets of AURORA 2 with multi-condition training for the ETSI repetition and the two adaptation approaches.

The total decoding time (in minutes) measured by decoding all of the three test data sets (set A, set B, and set C) of the AURORA 2 for the ETSI repetition and the two adaptation approaches with clean condition training and multi-condition training is shown in Figs 13 and 14, respectively. The decoding time was gauged on a personal computer with dual Intel Xeon E5-2690 CPU of 2.90 GHz and random access memory of 16 GB. No multi-thread processing was employed and the decoding program was executed sequentially. The platform used in the experiments was 64-bit Windows 10 Education. The experimental results show that the decoding time for the two adaptation approaches is much less than that of the ETSI repetition concealment method. That means if we employ any one adaptation approach, the same back-end server is capable of serving much more client users as compared with the ETSI repetition standards.

As we can see from **Table 1**, for multi-condition training and for a frame reduction factor of *D* = 2 as an example, even the word accuracy obtained with the proposed connecting-then-adapting approach is 0.3% (in average) worse than that obtained with ETSI repetition. However, from **Fig 14**, we find that using the proposed connecting-then-adapting approach, it allows the same back-end server to serve about twice the amount of client users without any extra cost of setting up new equipment as compared with ETSI repetition. Therefore, we can observe an appealing consequence of using the proposed connecting-then-adapting approach at the back-end server that the price paid (performance degradation) is small but the gain (computation cost saving) is huge.

## Conclusions

In this paper, we presented a new HMM adaptation approach that first connects the HMMs and then adapts the combined HMM for the recognition of RFR continuous speech. This new approach avoids the problems associated with creating transition links that skip too many states and violate the skipping length constraint. Therefore, it can remedy the insertion-prone problem caused by the old adapting-then-connecting approach. In our new approach, the destinations and associated probabilities that an emitting state may reach at the next RFR observation time are exactly the same as those it may reach at the next *D* FFR observation time in the FFR network. That is, at the same time, the (prior) probability of the emitting states in the RFR network is the same as that in the FFR network. We derived the formula for computing the transition matrix of the frame-rate-adapted HMMs and for computing the transition matrix of an HMM obtained by concatenating HMMs. We described the design and implementation of the old and new adaptation methods in detail and conducted experiments to compare and analyze the performance of the two adaptation approaches. The experimental results show that our new connecting-then-adapting approach can reduce the insertion error rate and obtain a slightly better accuracy than the adapting-then-connecting approach.

## References

[1] Tan Z-H, Varga I. Network, Distributed and Embedded Speech Recognition: An Overview In: Tan Z-H, Lindberg B, editors. Automatic Speech Recognition on Mobile Devices and over Communication Networks. London: Springer; 2008.

[2] Peinado A Speech Recognition over Digital Channels: Robustness and Standards: John Wiley & Sons; 2006.

[3] ETSI. ETSI ES 201 108 V1.1.3 (2003–09) Speech Processing, Transmission and Quality Aspects(STQ); Distributed speech recognition; Front-end feature extraction algorithm; Compression algorithms 2003.

[4] ETSI. ETSI ES 202 211 V1.1.1 (2003–11) Speech Processing, Transmission and Quality Aspects (STQ); Distributed speech recognition; Extended front-end feature extraction algorithm; Compression algorithms; Back-end speech reconstruction algorithm; Front-end extension for tonal language recognition and speech reconstruction 2003.

[5] Lee L-M, Jean F-R. Adaptation of hidden Markov models for recognizing speech of reduced frame rate. IEEE Transactions on Cybernetics. 2013;43(6):2114–21. 10.1109/TCYB.2013.2240450 23757520

[6] Tan Z-H, Dalsgaard P, Lindberg B, editors. Adaptive multi-frame-rate scheme for distributed speech recognition based on a half frame-rate front-end. 2005 IEEE 7th Workshop on Multimedia Signal Processing; 2005.

[7] Deng H, O'Shaughnessy D, Dahan J, Ganong WF. Interpolative variable frame rate transmission of speech features for distributed speech recognition. IEEE Workshop on Automatic Speech Recognition & Understanding 2007. p. 591–5.

[8] Lee L-M. Adaptation of hidden Markov models for half frame rate observations. Electronics Letters. 2010;46(10):723–4. 10.1049/el.2010.0942

[9] Lee L-M, Le H-H, Jean F-R. Improved hidden Markov model adaptation method for reduced frame rate speech recognition. Electronics Letters. 2017;53(14):962–4.

[10] Young S, Evermann G, Gales M, Hain T, Kershaw D, Liu X, et al The HTK book (for HTK version 3.4) Cambridge, U.K.: Cambridge Univ. Eng. Dept; 2006.

[11] Hirsch H-G, Pearce D, editors. The Aurora experimental framework for the performance evaluation of speech recognition systems under noisy conditions. ASR2000-Automatic Speech Recognition: Challenges for the new Millenium ISCA Tutorial and Research Workshop (ITRW); 2000.

[12] Young S, Russell N, Thornton J. Token Passing: A simple conceptual model for connected speech recognition systems Cambridge University Engineering Department Technical Report CUED F-INFENG/TR. 38, 1989.

[13] Tan Z-H, Dalsgaard P, Lindberg B. Automatic speech recognition over error-prone wireless networks. Speech Communication. 2005;47:220–242.

